# Medical Items and News of Interest to the Physician

**Published:** 1878-04

**Authors:** 


### Medical Items and News,

### For the Use of Physicians.

CULLED FROM THE STANDARD MEDICAL JOURNALS OF THE WORLD.

NOTE.—These formulæ are intended only for the regular practitioner of medicine, and should be employed only by him, or under his immediate supervision.

**Hydrophobia.**

Dr. J. G. S. Coghill reports two cases of hydrophobia treated by him in China, and reviewing the symptoms, he suggests that jaborandi might be a valuable remedy.

**Iodized Cod Liver Oil.**

R

Cod liver oil, 100 grammes.
Iodoform, 0 gramme 25 centigrammes.
Essence of anise, 10 drops.

M.

**Nausea and Vomiting.**

Dr. V. Jagielski has an article in the *British Medical Joarnal* on the value of koumys in affections where other food will not be retained by the stomach, and cites a number of cases to illustrate the success following its use.

**Cholera.**

R

Tinct. valerian. æther, 2 fl. ʒ.

Vin. ipecac, 1 fl. ʒ.

Aceti opii. 20 min.

Ol. menth. pip. 5 drops.

One dose: To allay the violent vomiting in cholera.

**Asthma.**

*The Clinic* reports an obstinate case of asthma which was entirely relieved by a two-grain pill of Grindelia robusta given three times daily. The medicine was thought to be the cause of a violent diarrhœa, which came on at the end of three weeks.

**Testing the Purity of Chloroform.**

Dr. Leucke, of Strasburg, gives the following simple method of testing the purity of chloroform: "Immerse a small piece of thin white blotting paper into the chloroform, and then let it dry in the air. As soon as the chloroform has evaporated, the paper will not present the least smell if the chloroform is pure. If there is any rancid smell perceptible, it indicates the presence of butyric acid in the chloroform, and as a rule is the strong characteristic of that substance."—*Philadelphia Med. Times.*

**Eucalyptus as a Local Anæsthetic.**

Dr. Horton (Ohio State Dental Society) speaks of the extract of eucalyptus as producing good results as a pain obtunder in sensitive dentine. A drop on a pledget of cotton is used. He thinks it the best of the local applications.—*Louisville Medical News.*

**Chloral dangerous in Delirium Tremens.**

In Naphey's *Medical Therapeutics* it is said: "It has been shown beyond reasonable doubt, by Dr. Madison Marsh, of Louisiana, and later by Dr. Ernest Magnan, of Paris, that drunkards do not bear chloral at all well. Its use by them, even in moderate doses, is liable to be followed by sudden death."

**Boils.**

A very simple remedy is made known by Dr. Sieven, in a St. Petersburg journal, for preventing the developement of boils. He states that if the skin be superficially scraped with a small knife, so that a drop or two of blood may be pressed through the epidermis as soon as the peculiar stabbing or pricking sensation and slight induration announce the commencement of the boil, it will not be further developed.

**Total Abstinence.**

Dr. Brudenell Carter strongly opposes total abstinence, and states that at three separate periods of his life he has tried abstaining from alcohol for three or four months, with the uniform result of a distinct deterioration of his health and working-power. He further states that he has seen life maintained for several months on alcoholic drinks alone; also that the writings of the total abstainers are sufficient evidence to his mind that in most cases their cerebral centers are not in a good state of vigor.

**Growth of Hair after Death.**

Dr. Caldwell, of Iowa, states that in 1862 he was present at the exhumation of a body which had been buried two years before. The coffin had sprung open at the joints, and the hair protruded through the openings. On opening the coffin the hair of the head was found to measure eighteen inches, the whiskers eight inches, and the hair on the breast five to six inches. The man had been shaved before being buried. In 1847 a similar circumstance occurred in Mercer county, Penn. In digging a grave, the workmen came upon the skeleton of a man that had been buried ten years. The hair was as firm as during life, and had grown to a length of eleven or twelve inches.

**Vance's Cream for Chilblains.**

Ointment of mercuric nitrate ℥ j.

Camphor ʒ j.

Oil of turpentine, ʒ ij.

Oil of olives, ʒ iv.

Mix well. To be applied with gentle frictions before the chilblains break.

---

**Death from Chewing Tobacco.**

A boy was recently admitted into the Melbourne Hospital suffering from the effects of chewing tobacco. He was in a very weak state, and complained of severe headache and dysentery. He became gradually unconscious and partially paralyzed, and died on the following day. The cause of death was narcotic poisoning.

---

**Foreign Bodies in the Throat.**

A British naval surgeon, Dr. Beveridge, states that for foreign bodies in the throat, such as pieces of meat, etc., a simple mode of relief is to blow forcibly into the ear. This excites powerful reflex action, during which the foreign body is expelled from the trachea, The plan is so easy of execution that if there is anything in it, it ought to be generally known and applied.

---

**Cough.**

For the irritating cough that is so harrassing to many patients, nothing is better than frequent small draughts of warm water; and when the throat is inflamed, or the tonsils swollen so that deglutition is painful, a wet napkin covered with a dry cloth should be put around the neck and worn until relieved, rewetting the cloth as often as it becomes dry. It should be wet in cool water.—*Med. Brief.*

---

**Migraine.**

Dr. Fort recommends the following:

℞.
Quinæ sulph, grs. xv.
Pulv. belladonnæ, grs. iv.
Ext. digitalis, grs. vijss.
Ext. Valerianæ, grs. xv.
Melis q. s. ut f. pil. xx.

Commenceing four days before the time when the paroxysm is expected, the pills are to be taken in this manner, viz: beginning with one in the morning and one at bedtime, the next day two are to be taken in the morning and one at bed-time. On the following day three are to be taken in the morning and a like number, at night, and on the day preceding the expected attack four are to be taken in the morning and five in the evening. This is to be repeated in a similar way in advance of subsequent attacks.

**Injection of Liquids into the Bladder.**

At the same meeting of the Society, M. Duchaussoy alluded to a communication in one of the journals in which the question was asked, "Is it possible to introduce liquids into the bladder without the aid of a catheter?" In reply, Duchaussoy asserted that nothing is easier. A caoutchouc bag of eight or ten ounces' capacity, provided with a tube terminating in a cone-shaped nozzle, can be introduced into the meatus, and with very moderate pressure the amount of fluid necessary can be forced into the bladder. The method is useful when the urethra is greatly inflamed.

---

**Gonorrhœa.**

M. Vidal has introduced into France the use of gurjun oil. He finds it an excellent substitute for copaiba, and in a recent thesis M. Deval corroborates this by a number of cases observed in M. Vidal's own clinic, and some others in M. Mauriac's. The dose advised should not exceed one drachm in the twenty-four hours. This amount will keep the bowels freely open. It is mixed with gum and some infusion. Its action is more rapid than that of copaiba, and it does not taint the breath. It may be used locally in vaginitis, etc. It has not provoked any eruption. M. Vidal has also employed it in leprosy.—*The Doctor.*

---

**Digestion of Cod-Liver Oil.**

One difficulty has always been felt, and it is this: even cod-liver oil is not always digested, and therefore something else was wanting. Dr. Balthazar Foster, of Birmingham, conceived the idea of utilizing Bernard's hint, and so combined ether with cod-liver oil. The increased flow of pancreatic juice so induced, led to the assimilation of the cod-liver oil, and thus another step forward was made in practical therapeutics. Another effect noticed by Dr. Foster, was the return of a liking for fat, under this kind of treatment, where previously a strong distaste to it had existed. One method is to give from ten to thirty drops ether (sulphuric) in the dose of oil; or the ether may be given in water immediately before the oil. In private practice Dr. Foster prefers to give the following mixture:

Potasse bicarb., ʒ jss–ʒ ij.

Acidi hydrocyan. dil., Mm, xij–xvj.

Spt. ætheris, ʒ jss–ʒ iij.

Aq. ad., ℥ viij. Misce.

℥ j ter in die sumat.

This method of adding to the usefulness of a course of cod-liver oil deserves wide and general attention.—*Dr. Fothergill's Hand-book of Treatment.*

**Prickly Heat.**

A "frequent contributor" to the *Indian Medical Gazette*, states that he has obtained most excellent results from the internal administration of liquor arsenicalis in prickly heat. He gives the remedy in the ordinary doses and with the usual precautions. This is a most tormenting affection to many persons in the hot months. It has been our misfortune to suffer from it for now a good many years, and hitherto we have found nothing of any particular use except cool weather. If this recommendation be well founded, it will be one of the blessings that occasionally come to us and make our daily life more tolerable.

---

**Obstinate Diarrhœa.**

Dr. Bonama, of Nantes, relates some cases confirmatory of the great and speedy utility of oxide of zinc in obstinate diarrhœa that has resisted various other remedies. He employs the formula recommended by Prof. Gubler, who first used the remedy for this purpose—viz., three grammes and a half (fifty-three grains) of the oxide, combined with half a gramme (eight grains) of bicarbonate of soda, and divided into three or four doses—one to be taken every three or four hours. The addition of the soda prevents the production of vomiting by the zinc.—*Bull. de Therap.*

---

**Living Insects in the Ear.**

The *Archives Medicales Belges* relates the following case: A little girl, three years old, put an insect called ladybird into her ear. Sharp cries, agitations and convulsive symptoms ensued. Injections of water were made without result. The physician then conceived the idea of asphyxiating the insect by means of chloroform. He dropped four drops of chloroform upon a small piece of cotton, which he introduced into the ear. Immediately the child ceased crying, and complained no further of any disagreeable sensation; the insect had become asphyxiated. An injection of warm water brought it away dead and no further trouble ensued.

---

**New Counter Irritant.**

It is related by Dr. Coutivier, in *L' Union Medicale*, that the extract of pimento is a most admirable revulsive in a large number of cases, and may usefully take the place of mustard and flies. It is proposed by M. Lardy. The writer says it acts with great rapidity, ten to thirty minutes, according to the point of application and the delicacy of the skin. Its action is manifested at first by a sensation of heat, a slight smarting and redness. These go on increasing for about three hours, then they remain stationary, and the revulsive action is so continued as long as may be desired. Nevertheless, after twenty or twenty-four hours in the adult, eight to ten in children, it is better to remove the plaster, and put another alongside of it, if it be desirable to continue the revulsion. The heat and tingling produced are painless and free from itching. . . . . The extract of pimento has a beautiful red color, identical with that of the dried fruit. Suitably incorporated in a plastic mass, and spread upon squares of paper, its application is very easy. It is unnecessary to warm it, for it adheres sufficiently to the skin; but it is well, on parts subject to movement, to fix it with a bandage just as a blister. Moreover, its action may be augmented or moderated according to the pressure. On removal, the heat and tingling may be immediately arrested by the application of a little starch.

---

**Typhoid Fever.**

In the *British Med. Jour.* Dr. Persse White gives the experience which he has had with the employment of turpentine in cases of typhoid fever complicated with bronchitis or diarrhœa, and mentions the following formula as having furnished good results:

℞

Olei terebinthinæ, ʒ ij.

Liq. potassæ ʒ ij.

Mucilaginis acaciæ, ʒ iv.

Syr. papaveris, alb.

Syr. flor. aurantii, āā ʒ viij.

Aq. Camphoræ, q. s. ad ℥ viij.

Fiat Mistura. A tablespoonful to be taken every fourth hour, the bottle being first shaken.

---

**To Blister the Skin Quickly.**

Into a watch-glass, pill-box, or any similar small receptacle, pour ten drops of concentrated water of ammonia (aqua ammonia fortior); cover the liquid with a bit of linen or a little cotton wool, and at once apply the cup to the skin where the blister is required. Press so that the vapor is confined to the inside of the vessel. A red circle will directly be observed outside, when it will be certain vesication has taken place. Half a minute or so is all the time required to obtain the result. The blister may be dressed in the usual manner of dealing with a blister from cantharides. Acetic acid, concentrated, applied to the skin will also in a few minutes produce vesication. In each case evaporation should be prevented by some suitable covering. Bibulous paper slightly wetted with a little of ethereal extract of cantharides, instantly applied to the skin, and covered with a piece of adhesive plaster, will answer for the same purpose.

**Ciliary Neuralgia.**

Croton chloral seems to have an almost specific influence on the sensory fibres of the fifth nerve, and it can be more surely relied upon to allay those fearful pains which attend the violent inflammation of the iris and choroid, and are known as ciliary neuralgia. In all cases in which it was given for this neuralgia it has exerted its anæsthetizing effect without producing any collateral disturbance. This is the formula:

℞

| | |
|---|---|
| Croton-chloral hydrate, | 1 gramme. |
| Spr. vin. rectif. | 4 " |
| Aq. destill. | 150 " |
| Syr. aurant. cort. | 15 " |

M. 1 tablespoonful every two hours.—FRIEDINGER, in *Wiener Med. Wochenschrift.*

---

**Bright's Disease in Apparently Healthy Subjects.**

At a recent meeting of the Kings County Medical Society (N. Y.), B. A. Segur, M. D., read an exhaustive paper on the "Prevalence of Bright's Disease and its Discovery in apparently Healthy Subjects," which will bear a very careful perusal, but, consisting in the main of statistics, cannot be condensed. His two conclusions of practical value are, that a large proportion of deaths, recorded under the names of pneumonia, pleurisy, pericarditis, apoplexy, convulsions, diarrhœa, etc., have Bright's disease for their predisposing cause, and that, while the appearance of albumen in the urine marks an advanced stage of kidney degeneration, the hyaline cast is a very early and important means of diagnosis, as significant in its relation to the kidney as the mucous rale is in its relation to the lungs.

---

**Danger of Ether.**

The following painful occurrence is related by the Paris correspondent of the New York *Times:* Whatever may be the dangers attending the application of chloroform, it has, at least, the advantage of not being inflammable.

A young lady of eighteen, remarkably beautiful, belonging to a family of rich merchants of Lyons, had to undergo a surgical operation. The surgeon said it was necessary to give her ether. The sack was prepared, and the young lady had been inhaling it for a moment, when a light was brought near the patient. In an instant the ether was ignited, and the sack exploded. The doctor was himself seriously burned, but the young lady was in a lamentable condition. Her nose was taken off completely, and one side of the upper jaw was laid bare. It is needless to say that she is horribly disfigured for life. No one could describe the despair of the family, and perhaps it would have been better had the poor girl died from the effects of this dreadful wound. It is rumored that the doctor has committed suicide.

---

**Needle found in the Brain.**

At a meeting of the Pathological Society of Philadelphia (*Medical Times*), Dr. H. L. Hodge reported that upon removing the calvaria of a subject in the anatomical rooms of the University of Pennsylvania, a sewing-needle of medium size was found lying on the right hemisphere of the brain, nearly parallel to the superior longitudinal sinus, about one inch distant from it, and about an inch and a half behind the fronto-parietal suture. The point and the eye of needle were both unbroken. The point was directed backward. The needle was much oxidized, and attached to the arachnoid surface of the dura mater by old bands of lymph near the larger extremity of the needle. No history of the cadaver, an adult male, could be obtained. The needle appears to have given rise to no important changes, and had no apparent connection with the cause of death. The man seems to have died of phthisis.

---

**To Lessen Quantity of Quinine.**

In a lecture published in the *Medical Record*, Prof. Thompson says:

Capsicum combined with quinine will diminish the size of the dose requisite, and the same may be said of ginger and other aromatics. A good dose of capsicum combined with twenty grains of quinine will act as well as thirty grains of quinine without the capsicum. Spices in general stimulate the portal circulation and promote the flow of bile, and hence their universal use in hot climates. There is a tendency on the part of quinine and capsicum to purge, and sometimes to purge violently. In such cases the purgative action is caused by the increased flow of bile produced by the capsicum. Ginger and quinine, when combined, do not purge, and it makes a very good combination. If the medicine is administered in the form of pills, capsicum may be preferable, because of the less bulk required; but, if desirable, the ginger may be given separately, and with the same effect as when combined with the quinine. The proportions should be one grain of capsicum to three of quinine; with ginger, one grain of each.

[It may be asked as a conundrum why, to procure equal effects, quinine is now required in doses so much larger than those given for some years after its discovery. A few grains then did as much work as half a drachm will at present.]

**Epilepsy.**

Dr. Jas. B. Ayer reports (*British Medical and Surgical Journal*) twelve cases treated by the following prescription for two years: [Brown-Sequard's.]

℞ Sodii bromidi, potassii bromidi, ammonii bromidi, āā ʒ iij; potassii iodidi, ammonii iodidi, āā ʒ iss; ammoniæ sesqui-carb., ʒ i; tinct. calumbæ, f ℥ iss; aquæ destillat. ad f ℥ viij. M.

Full dose, one and a half drachms before each meal, and three drachms at bedtime.

*Results.*—In *four* cases very satisfactory: reduced to a single attack in forty-six months, thirty-one months, twenty-two months, and sixteen months, respectively. In *five* cases number and severity of attacks both diminished. In *one* case severity diminished, numbered unchanged. In *two* cases no change in number or severity. In *eleven* cases there has been *marked* improvement in general health and mental condition. In *one* case there has been a *slight* improvement.

---

**Orchitis.**

Dr. Julian Alvarez, in an article contributed to the Spanish Journal, *La Independencia Medica*, gives several cases in which he has tried iodoform. He say that it alleviates the pain in blennorrhagic orchitis by reason of its anæsthetic properties, in this respect its action being superior to belladonna, cicutine, opium, or other alkaloids; that this remedy, in consequence of its richness in iodine, exerts its antiphlogistic and resolvent action without any inconvenience to the patient, which is in striking contrast with the action of mercury. Iodoform, moreover, materially shortens the duration of blennorrhagic orchitis, while it avoids the danger of consecutive induration of the testis. The ointment that ought to be employed should contain one to two parts of iodoform to thirty of the excipient, according to the intensity of the inflammation. Dr. Alvarez states that in none of the cases in which he employed this remedy did he observe any eruption of the skin, so common when other ointments are used.

---

**Death from a Carious Tooth.**

A singular case of death lately took place at the Lariboissière Hospital, under the following circumstances: The deceased, a boy aged seven, was admitted into the hospital complaining of pain in the head accompanied with a tendency to stupor. His eyes were somewhat prominent, but nothing else remarkable was noticed in the patient. About a month after admission coma set in, and this was accompanied with high temperature, which carried him off. A small, hard body was felt over the right eye, which was supposed to have been the cause of death. At the post-mortem examination the real cause was traced to a carious tooth, one of the inferior molars, which caused an abscess in the jaw. The inflammatory process extended along the dental nerve, entered the skull through the orbit, involving the dura mater and the brain. The latter contained two or three abscesses, and the bones of the skull were necrosed to a considerable extent. An abcess was also found in the heart. This case would show how important it is to attend early to carious teeth, and to have them removed as soon as inflammatory symptoms become evident, and not to tamper with them by "stopping," or other useless means frequently resorted to by dentists, to the detriment of the patient's pockets, if not of their health or life.—*British Medical Journal.*

---

**Foreign Bodies in the Stomach.**

"L'homme a la fourchette," so famous in Paris a year or two ago, is distanced by a man in Australia now undergoing imprisonment for being unable to restore a gold ring which he had swallowed, belonging to the prosecutor. He is being treated by the visiting surgeon of the jail, with the view of making him disgorge a large steel Albert chain and a common brass ring. The chain can be distinctly felt at the bottom of the stomach, and the prisoner states that it is now nine months since he swallowed it, and it is the only one he has had any difficulty about. He says that he has had two pounds weight of jewelry in his stomach, and has had watches there as long as twenty-four hours. The jailer has a collection of objects, such as Albert chains, penknives and rings, which he has procured by making him vomit by emetics. The prisoner is an intelligent young man of twenty-three—*Med. and Surg. Rep.*

---

**Pathological Sweating.**

M. Royet, in his thesis, furnishes the results of the trials made with sulphate of atropia by Professor Vulpian since 1873. These demonstrate the efficacy of atropia in sweating under the most various circumstances—as phthisis, rheumatism, convalescence, prolonged suppuration, hysteria, and the influence of jaborandi. The dose of the sulphate varies from half a milligramme to one and a half, it being very rarely desirable to go beyond this. The most convenient form to administer it is in pills or granules, each containing half a milligramme. In order to act with efficacy, the medicine should be given a few hours prior to the occurrence of sweating. Thus, in the nocturnal sweating of phthisis, the pill should be given at eight or ten o'clock in the evening. At least two hours should elapse between the doses, and, if two or three are required in the twenty-four hours, these should be divided by equal intervals. From two to four days suffice to produce a suppression or notable diminution of the sweats; but, in order that the effect may be durable, the use of the atropia should be prolonged, with some diminution of the dose, for eight or ten days. The author of the thesis agrees with Professor Vulpian in believing that it is nowise imprudent to suppress sweating in rheumatism.—*Lyon Medical.*

**Glycosuria.**

Dr. Garnier records a case of glycosuria which was treated very successfully by carbolic acid. Equal parts of carbolic acid and alcohol were mixed, and of this solution two minims were given for a dose. At no time during the treatment did the dose of the acid exceed six minims per diem. He also records a case of puerperal eclampsia occurring before parturition, due to uræmic poisoning, which he treated successfully with carbolic acid.

Dr. H. Fischer, of Breslau, administers carbolic acid to diabetic subjects before surgical operations. The carbolic acid treatment in diabetes was first recommended by Ebstein and Muller. Fischer reports that if carbolic acid be given in small and frequently repeated doses, it will in a short time cause a considerable reduction in the amount of sugar in the urine, and permit the surgeon to operate with the ordinary chances of success. The use of the carbolic acid should be kept up during the after-treatment, and not be discontinued until the wound be quite closed.—*Medical and Surgical Reporter.*

**Intestinal Gas and Flatulent Dyspepsia.**

The following conclusions are drawn from a paper upon the above subject, read by M. Leven before the Academy of Medicine, Paris:

1. Food does not seem to produce gas; that found in the digestive tube comes from the external air, the blood, and the fæcal matters.

2. The gas formed during the course of flatulent dyspepsia is not due to the decomposition of food, but comes from the three above mentioned scources. It is continually kept in motion by the pathological contraction of the intestinal muscular fibre. Although continually expelled, it is constantly renewed. Its production may be incessant, as well in the fasting individual as in one well nourished.

3. This symptom, production of gas, therefore implies the existence of an intestinal irritation which is always consecutive to a stomachal dyspepsia of already long duration.

4. The course of the disease, and the treatment followed to obtain a cure, confirm these clinical observations.

5. There is no necessity of instituting a medication against the gas *per se;* moreover, the so-called absorbent powders, as charcoal, do not absorb the gas, as I have proved experimentally. Although block charcoal may possess this property, as soon as it is reduced to powder it loses it completely.—*Detroit Lancet.*

**Tearless Madness.**

One of the most curious facts connected with madness is the utter absence of tears among the insane. Whatever the form of madness, tears are conspicuous by their absence, as much in the depression of melancholia, or the excitement of mania, as in the utter apathy of dementia. If a patient in a lunatic asylum be discovered in tears, it will be found that it is either a patient commencing to recover or an emotional outbreak in an epileptic who is scarcely truly insane. While actually insane patients appear to have lost the power of weeping—it is only returning reason which can once more unloose the fountains of their tears.—Even when a lunatic is telling one in fervid language how she has been deprived of her children, or the outrages that have been perpetrated on herself, her eye is never even moist. The ready gush of tears which accompanies the plaint of the sane woman contrasts with the dry-eyed appeal of the lunatic. It would, indeed, seem that tears give relief to feelings which when pent up lead to madness. It is one of the privileges of reason to be able to weep. Amidst all the misery of the insane they can find no relief in tears.—*British Med. Journal.*

[We doubt the truthfulness of this theory; in fact, we know it is not correct, for we have seen insane people weep and shed tears too, repeatedly.—What is the matter with the *British Medical Journal*, we wonder?—ED. BISTOURY.]

**Puerperal Convulsions.**

Two years ago, Dr. O. S. Boyd's suggestions in regard to the treatment of puerperal convulsions with veratrum viride, were sharply criticised as being extra hazardous. In the January number of the *American Practitioner* he publishes a paper on the same subject, referring to ten cases treated by Dr. Fern of Brooklyn, and yet another which came within his own observation. The patient, a primipara, had four convulsions, at intervals of 30 to 75 minutes, and lasting from 5 to

7 minutes. A few minutes after the last one, consciousness having been recovered, 20 drops of the tincture of veratrum viride were given, and the dose was repeated every 15 minutes until 120 drops had been taken. Following the sixth dose the pulse had fallen from 144 to 130. Ten minutes after this dose had been taken, the patient vomited nearly a pint of tenacious mucus, colored by the veratrum. Within ten minutes after the vomiting began, the pulse was 54 per minute. Vomiting followed three times in quick succession, and she was then given 25 drops of laudanum, and this was repeated at every subsequent attack of vomiting, which amounted to four times in all. A quiet sleep then came on. Directions were given to resort to the veratrum whenever the pulse rose to 80, and two doses only at long intervals had to be given. The patient made a good recovery. Dr. Boyd believes that the use of veratrum will come to be considered no more hazardous than that of ipecacuanha, and much less dangerous than Lobelia in flata; and that it will entirely supersede the resort to blood-letting.

---

**Hypodermic Use of Ether in Labor.**

The following case is recorded in the *Medical Press and Circular*, by A. V. Macan, M. B., Assistant Physician to the Rotunda Hospital, and Obstetric Surgeon to the City of Dublin Hospital.

The patient, who was pregnant for the eleventh time, had had good health till within nine weeks of her confinement. She then began to complain of a gnawing pain in the lumbar and hypogastric regions. The abdomen was much larger than in any of her former pregnancies, which was caused by hydrops amnii. The amount of urine passed was much below the normal quantity. The labor was very tedious, from uterine inertia. The membranes were therefore ruptured, when an immense quantity of liq. amnii escaped. The fœtus belongs to the class of anencephalous monsters, the diagnosis resting chiefly on the unusual shape of the mastoid processes, and the violent movement of the fœtus when the finger came in contact with the exposed portion of the medulla oblongata. It was born without artificial assistance, but its birth was followed by post partum hæmorrhage and retention of the placenta. This necessitated the manual removal of the placenta, which was accompanied with but slight additional loss of blood. The woman, however, got gradually worse, exhibiting all the symptoms consequent on severe loss of blood. I therefore determined to try the effect of the subcutaneous injection of ether, as recommended by Professor v. Hecker, of Munich.

As some blood was still escaping *per vaginam*, I thought it necessary to combine it with the injection of the perchloride of iron into the uterus. Soon after 44 ♏ of ether had been injected well into the cellular tissue of the abdominal walls, reaction suddenly set in. The change was so sudden and unusual that no doubt could be entertained that it was due to the ether. The woman's convalescence was rapid and uninterrupted, she being able to leave her bed on the twelfth day.

The chief point to be attended to in making the injection is to pass the syringe well down in the subcutaneous cellular tissue, otherwise troublesome abscesses may form at the seat of the injection. The quantity to be injected depends entirely on the pulse. Professor v. Hecker frequently injects from two to four drachms at short intervals. The effect is very transient, so that the injection may have to be repeated. Its use need not be confined to collapse from post partum hæmorrhage. I have also tried it in accidental hæmorrhage, rupture of the uterus, and puerperal fever, in all cases with more or less effect. Dr. Atthill, the present Master of the hospital, has used it with good effect in a case of placenta prævia, and it has been used by Dr. Bennett and Dr. Croly for collapse in cases of strangulated hernia. Professor Winckel, of Dresden, has used it in a case of pulmonary embolism following delivery, where it completely relieved the intense paroxysms of dyspnœa.

---

**Home-Made Mineral Waters.**

A writer in the *Medical Press and Circular* says: At my instigation, some of my medical friends have used the following mixture where the bitter saline purgative waters of Friedrichshall and Hunyadi Janos were indicated, with equal if not more satisfactory results in abdominal diseases, hepatic congestion, even attended with hæmorrhoids, plethora, etc.:

Sulphate of soda, 3 drachms.
" potassa, 3 drachms.
" magnesia, 4 drachms.
Bicarbonate of soda, 1 drachm.
" potassa, 1 scruple,
Water, 20 ounces,
Muriatic acid, 1 drachm.

Mix. The bottle is to be kept well corked, and in a cool place.

A wineglassful the first thing every morning, in a tumbler of cold water. The addition of the muriatic acid answers a twofold purpose; it saturates the mixture with carbonic acid gas, making it more palatable, and the small quantities of chlorides it generates add to its efficacy in a surprising way. Sulphate of potassa is the best cholagogue in the saline shape, and invariably enters largely into all the natural waters of use in hepatic congestion. But all the natural waters contain more or less sulphate of lime (in common *parlance*, plaster of Paris), which adds nothing to its efficacy, and is objectionable.

Again: In gouty and rheumatic diatheses, where an iodized alkaline aperient is indicated, the following may be prescribed, and will be found far more efficacious than any of the natural waters:

Dry sulphate of soda, 3 ounces.

" " " potassa, 6 drachms.

Bicarbonate of potassa, 2½ drachms.

Carbonate of lithia, ½ drachm.

Iodide of potassium, ½ drachm.

Mix. Dose, a teaspoonful the first thing in the morning in half a pint of tepid water.

If the patient prefer cold to tepid water, plain cold, or aerated may be used.

In renal affections, where a course of the warm alkaline waters of Vichy or Carlsbad, or the cold ones of Vals, Jachingen, and Marienbad is desired, we may use (as Dr. Wade suggests) dilute solutions of potassa and soda bicarbonate with citrate of lithia. Sir H. Page has found soft or distilled water of great service in the palliative treatment of renal affections, and either the one or the other should always be used in the preparation of the solutions. They may be taken warm, or charged with carbonic acid.

By adopting such measures as these, we can confer, in some measure, the boon of mineral waters on the poor patients, which is now only enjoyed by the wealthy.

---

**Scrofulous Diseases.**

In the multitude of new remedies which the wonderful activity of persons engaged in preparing or in prescribing medicines is constantly bringing to light, we find, occasionally, therapeutical agents, that the present generation has allowed to become obsolete or forgotten, are raked from the dust that covers them, and are recommended anew for the readiness with which they modify or cure diseases of the profoundest constitutional nature. The muriate of lime, as it was called by the earlier physicians, has recently been attracting a good deal of attention in Britain, and, as may be seen, it already has its advocates on this side of the Atlantic.

The following paragraphs are taken from a late number of the Philadelphia *Medical and Surgical Reporter*, and were furnished for publication by Dr. A. H. Mellersh, resident physican to the German Hospital of Philadelphia. It ought to be noted that common bleaching powder is not the proper salt to be used. The liquor calcii chloridi of the Pharmacopœia is what is indicated. Dr. Robert Bell strongly recommended it in the London *Lancet* of last August for tuberculosis and wasting diseases of childhood, and Dr. Coghill, in the London *Practitioner* of October last, declares he has found it to be of great value in many strumous affections. It is advised that the medicine shall be given in milk or syrup.

Dr. Mellersh reports:

E. M., a girl of thirteen years, a strumous subject, has been suffering for two years with chronic diarrhœa and tumid abdomen; both lungs are tuberculous, the apices being largely involved; she was reduced to almost the last degree of emaciation. Has been under the usual treatment of cod-liver oil, tincture of bark, syrup of the iodide of iron, with milk diet, etc. She was not able to leave the bed.

After taking the calcium chloride the diarrhœa stopped, and there was a decided improvement, so much so that in the course of two weeks she was able to walk to the dispensary, a distance of six blocks.

In a number of phthisis pulmonalis cases, both in and out of the hospital, the remedy has appeared to act beneficially, and in several cases the patients have asked for the remedy after it had been discontinued.

In a case of typhoid fever, under Dr. J. S. Cohen's charge, after other remedies had failed, the calcium chloride relieved the diarrhœa at once.

In some of the chronic and acute diarrhœas of children it has appeared to me to be very salutary in its effects, and I shall be glad to hear the results of more extended trials in this direction.

The cheapness and innoxious character of the drug also recommend it, especially in dispensary practice; the solution is best given in thirty-drop doses, in milk, for an adult. For children it may substitute the stereotyped aqua calcis and milk, or it may be given in emulsion with cod-liver oil.

It is not claimed that calcium chloride has any remarkably specific action, but, from the limited experience I have had, I judge that it owes its virtues to its salutary effect upon the assimilative functions of the alimentary canal, and to this is attributable its beneficial action in somewhat dissimilar diseases.

It is also well known that tubercle undergoes calcareous degeneration, and that this change in the deposit is favorable, and, in fact, that convalescence in tubercular subjects is sometimes effected by this metamorphosis. Is it possible that by supplying the calcium we encourage this mode of recovery? The close chemical relation with sodium chloride gives the idea of its easy assimilation and general use.

**Formulæ for Preparing Lister's Antiseptic Medicaments used in Surgical Operations.**

The new method of dealing with surgical cases, recently brought into vogue by Mr. Lister, an eminent Scotch Surgeon, has been reported on by Dr. A. C. Girard to the Surgeon General of the U. S. Army, and we extract from his report the processes of making the various articles employed in each case. Dr. Girard says this is a method of treating wounds based on the "germ theory," and its principal aim is to prevent the entrance of germs into wounds, to destroy them if already there, and to guard against the accumulations of wound-secretions. In order to attain this end he surrounds the patient with a series of precautionary measures, which have from time to time been improved on by him. "These require peculiar materials and appliances, of which I present the latest phase that came under my observation. They are enumerated without particular plan. Where the mode of preparation is not indicated, it will suggest itself; where it is described it is adapted as much as possible to self-preparation, thereby facilitating the introduction of the system by diminishing its greatest objection—the cost. Their use is described as concisely as possible, and will become clearer when I shall treat of the mode of operating and dressing.

1. CARBOLIZED SOLUTION (Acid. carbol. cryst. 5, aq. 100) is used to clean the neighborhood of wounds before operation, to disinfect the hands of surgeon and assistants, and instruments, to wash out septic wounds and clear drainage tubes.

2. CARBOLIZED WATER, a 2½ per cent solution of crystalized carbolic acid in water. It is used in the spray and to wet the "lost gauze."

3. CARBOLIZED OIL (Acid. carbol. cryst. 5, ol. oliv. 100), to oil catheters or other instruments, or fingers when about to be introduced into some of the cavities. It is also employed when a constant direct contact of the antiseptic with the wound is necessary, as in caries,—or where the gauze dressing cannot be applied, as in abscess of the rectum.

4. THE SPRAY. In order to prevent the entrance of living germs, during an operation or dressing, a spray of "carbolized water" is directed on the wound. The best instrument for this purpose is "Lister's spray," a steam atomizer which throws a large cone of finely divided spray. It is almost indispensable in long operations and where a considerable space of tissue is exposed. In its absence it may be replaced by the ordinary steam atomizer, of which two ought to be at hand, as they are sooner exhausted.

5. CATGUT is the *sine qua non* of Lister's dressing for ligatures and deep sutures. In a few days it is absorbed and the wounds close over it without danger of its acting as an irritant or permitting hæmorrhage. In order to prevent its becoming soft, spongy and slippery, it has to go through a process, by which it is rendered tough, flexible and able to resist the action of the wound-secretions for a sufficient length of time. It is first shaken up in a carbolized emulsion of 1 part cryst. carbolic acid (dissolved by addition of 10 per cent water), and 5 parts olive oil. It should then be no more disturbed but set aside in a cool place. The catgut should be kept separate from the watery part of the emulsion. This is most conveniently done by placing a sufficient number of pebbles into the vessels containing the emulsion to support a small disk of glass out of reach of the water, on which the coils of catgut are laid. It takes at least two months before it acquires the necessary qualities. The catgut is used in different sizes, from the thickuess of a horsehair to that of twine.

6. SALICYLIC COTTON. It is prepared by first removing the fatty matter of the cotton, by boiling it in lye or other alkaline liquid. Thus the cotton becomes hygroscopic, which quality may easily be tested by throwing a small ball on water. In its natural state it will not sink—if prepared it will go to the bottom of the vessels in a very short time. The proportions are about 8 ozs. of the acid, 8 ozs. alcohol, and 2 gallons of water. The addition of about 20 per cent of glycerine is said to prevent the disagreeable dusting of the acid.

7. SPONGES. All sponges used in connection with Lister's dressing, in the cleaning of wounds and absorption of wound-secretions, should be prepared in the following manner: After being beaten, cleaned and washed out in lukewarm distilled water, they are immersed in the "carbolized solution," and kept there until needed. After use they are washed out in the solution and replaced in the vessel. In this manner they can be used over again as long as they last.

8. DRAINAGE-TUBES are small flexible rubber tubes, of the size of a small quill to that of a little finger, with numerous openings on the sides, each of half the diameter of the tube. Their use is to facilitate the egress of wound-secretions without interfering with union.

**Chlorate of Potash a Specific in Diphtheria.**

Chlorate of potash was one of the earliest and continues to be one of the most common remedies in the treatment of this serious affection. But, after all, but little real confidence is felt by the practitioner who uses it, that it will exert much influence in arresting the progress of the disease. Many other curative means have been recommended. The columns of the BISTOURY in the previous numbers contain most of them. Some, no doubt, are good, but none seems by common experience to be held as really a specific. It may be that the dose is usually too feeble, or that it is not given sufficiently often to produce full constitutional action. At any rate, it too frequently falls short of what is expected of a really efficient medicament. Chlorate of potash has been resuscitated, one may say, by Dr. Seeligmuller, of Halle, (Prussia). He recounts in the London *Medical Times* his own wide experience as an hospital physician in treating diphtheria, and his wonderful success in speedily arresting its progress by employing a saturated solution of this salt. His testimony is that chlorate of potash is as much a specific in this disease as quinine is in intermittents. Topical application there is no objection to, but it is not at all necessary. It is useful only as it quiets parents and friends, and satisfies them that everything is being done by the physician that the exigency of the case demands. It saves the medical adviser from the suspicion of doing nothing when so much is required. Gargling, anointing, brushing and cauterizing is time-wasting to the physician and tedious to the patient. It may all be omitted without the physician accusing himself of neglect. The formula of Dr. Seeligmuller is as follows:

Chlorate of potash, 10 grammes.
Distilled water, 200 grammes.

Make a solution and give a teaspoonful every one or two hours to children above the age of three years. A syrup may be employed as a vehicle to improve the flavor; otherwise it may be omitted. The same solution may be ordered as a gargle. The strength is one of five per cent.

As long as there are any relics of the diphtheritic deposit, give the medicine every hour, later, if the case progresses favorably, every two hours; at the *beginning of treatment, day and night without intermission.*

For some minutes after the administration, drinks must be withheld, to avoid washing the medicine away too quickly from the fauces.

The first effect will be a disappearance of the fetid breath. The deposits will gradually diminish, and, above all, there will be a rapid amelioration of the general condition. "Children," the writer says, "whom I found in the evening when I was called in, reduced by exhaustion and fever, the next morning were sitting up in their beds fresh and without fever, demanding food and playing."

When there was great debility, stimulants were employed to strengthen the action of the heart, wine, beef tea, etc.

These large doses of chlorate of potash may diminish the energy of the heart, and they may disorder the stomach. If necessary the medicine should in such cases be withheld for a few hours, and when the serious symptoms have vanished it may be resumed. Dr. Seeligmuller concludes his report with the subjoined summary of his experience:

1. The chlorate of potash administered in a saturated solution (5 per cent.) has a specific effect on diphtheria.

2. It must be given in a solution of ten grammes in 200 grammes of distilled water, without adding any syrup or any other substance to ameliorate the taste.

3. This solution is to be ordered to infants under three years at half a spoonful every two hours (if the malady is very grave, every hour) at first *day and night* without interruption.

4. This internal medication *alone* will suffice in all cases.

5. The saturated solution of chlorate of potash exercises (*a*) a topical action, and (*b*) a general one on the diphtheritic process—(*a*) a topical one, as a mild cautery, and by separating the diptheritic pseudo-membranes from their basement membranes; (*b*) a general one, supplying the oxygen withdrawn from the blood corpuscles by bacteria and destroying these organisms.

6. Caution is required lest the saturated solution may act dangerously on heart or digestion. When such symptoms occur, the administration must be suspended.

It ought to be added, to give authority to the foregoing, that the chlorate was administered in the way directed, to a great number of both hospital and private patients, and that it was pursued through several years of time. There was uniform success.

**The Treatment of Ozæna.**

Dr. Rouge, in a paper read at the third meeting of the Congress, arrived at the following conclusions:

1. Ozæna is the result of suppuration in the nasal fossæ, or their annexes, viz., the frontal, maxillary and sphenoidal sinuses, and the ethmoidal cells.

2. The suppuration appears to originate in all cases in disease of the nasal fossæ, or their annexes.

3. The degree of fœtor of the air issuing from the nasal fossæ depends upon the extent of the osseous lesions which have given rise to the ozæna.

4. The latter is also increased by the stagnation of pus in the sinuses.

5. Should the surgeon fail to trace the disease to some affection of the nasal cavity, he must seek for it in the sinuses and the ethmoidal cells.

6. The local treatment of ozæna comprises:

(a.) Frequent washing out of the nasal cavity by injections, which vary with the special indications of each case. (b.) The insufflation of disinfecting, caustic, or astringent powders. (c.) Cauterization of various kinds; the use of the galvano-cautery. (d.) In severe cases all sequestra must be removed, and the sinuses completely drained. The nose is detached by the sub-labial method; this enables us to explore the nasal fossæ directly, to remove all necrosed portions of bone, and to open the sinuses. The formation of a cicatrix is thus avoided.

7. It is unnecessary to speak of any general mode of treatment, as this must be regulated according to the patient's constitution.

M. Verneuil said he had not applied the method of Dr. Rouge, but he would do so. He remembers that M. Torbat had some good results from it.

Dr. Ollier adopted this method in certain cases.

Dr. Rouge believed that ozæna is frequently due to alteration of the sinuses, but generally to caries of the ethmoidal cells. Posterior rhinoscopy is not so useful, because the mucous membrane is so swollen that we can not conveniently discover the diseased parts.—*Med. Press and Circ.*

---

**Pimply-Face Acne.**

In a recent lecture by Mr. Jonathan Hutchinson, an eminent London surgeon, in which he discusses the whole subject of this unsightly affection—its causes and appearances—he says in regard to the best treatment, as follows:

'When the face is covered with pimples, some of which are red, some contain pus, and others show only black points in their centres—all kinds being present, and all show in progress—it is commonly agreed to call the condition Acne.

The rules for the constitutional treatment of acne patients follow easily from what we have said. If the patient be young he should be made to use a cold bath every morning, to take plenty of exercise, to live liberally as regards meat diet, with a fair allowance of stimulants; and he should be cautioned or encouraged, as the case may be, in reference to sexual matters. As to medicines, a long course of small doses of arsenic will often be of great use. If constipation be present, the habitual use of a chalybeate aperient should be prescribed. You may do all this, however, most sedulously and gain nothing whatever, if you neglect local measures; whilst with the latter only, and without any change in the patient's habits, you may often get an acne eruption so nearly well that he will regard it gratefully as a cure. The chief local measure consists in destroying, by means of a fluid caustic, the inflamed follicles. With a fine-pointed glass brush, or a bit of soft wood cut to a point, you touch the inflamed spots from day to day. Take care not to apply too much. In the left hand should be a roll of blotting paper with which to absorb the fluid if it has been deposited too abundantly. The best fluid to use is the acid nitrate of mercury. It will usually be necessary to repeat the touching once a week for a month or two, carefully seeking out every fresh spot. After that the patient should still see you once a month, in order that the cure may be kept up. The acid thus used does not leave larger scars than the spots would themselves do.

In acne rosacea the use of the caustic will again serve an excellent purpose. You may not only touch the spots themselves, but also pencil out the stray vessels which add so much to the patient's disfigurement. He, or more usually she, will gladly exchange a few slight and scarcely perceptible scars for the angry and very suspicious-looking redness of face which the disease causes.—*Medical Times and Gazette.*

---

**Ergotine for Hypodermic Injections in Metrorrhagia.**

Ergotine, (de Bonjean), 2 grammes.
Glycerine.
Water, āā 15 grammes.

